# Effects of Microhabitat Temperature Variations on the Gut Microbiotas of Free-Living Hibernating Animals

**DOI:** 10.1128/spectrum.00433-23

**Published:** 2023-06-28

**Authors:** Sen Liu, Yanhong Xiao, Xufan Wang, Dongge Guo, Yanmei Wang, Ying Wang

**Affiliations:** a College of Life Sciences, Henan Normal University, Xinxiang, China; b Jilin Provincial Key Laboratory of Animal Resource Conservation and Utilization, Northeast Normal University, Changchun, China; Connecticut Agricultural Experiment Station

**Keywords:** temperature, gut microbiota, 16S rRNA gene sequencing, hibernation, *Rhinolophus pusillus*

## Abstract

Variations in ambient temperature (*T_a_*) may significantly influence the gut microbiotas of ectothermic and endothermic animals, affecting fitness. It remains unclear, however, whether temperature fluctuations affect the gut microbial communities of hibernating animals during torpor. To investigate temperature-induced changes in the gut microbiota during hibernation under entirely natural conditions, we took advantage of two adjacent but distinct populations of the least horseshoe bat (Rhinolophus pusillus), which inhabit sites with a similar summer *T_a_* but a different winter *T_a_*. Using 16S rRNA gene high-throughput sequencing, we estimated differences in gut microbial diversity and composition between the hibernating (winter) and active (summer) *R. pusillus* populations at both sites. During the active period, gut microbiotas did not differ significantly between the two populations, probably due to the similar *T_a_*s. However, during hibernation, a higher *T_a_* was associated with decreased α-diversity in the gut microbiome. During hibernation, temperature variation did not significantly affect the relative abundance of *Proteobacteria*, the dominant phylum at both sites, but marked site-specific differences were detected in the relative abundances of *Firmicutes*, *Actinobacteria*, and *Tenericutes*. In total, 74 amplicon sequence variants (ASVs) were significantly differentially abundant between the hibernating and active bat guts across the two sites; most of these ASVs were associated with the cooler site, and many belonged to pathogenic genera, suggesting that lower ambient temperatures during hibernation may increase the risk of pathogen proliferation in the host gut. Our findings help to clarify the mechanisms underlying the gut microbiota-driven adaptation of hibernating mammals to temperature changes.

**IMPORTANCE** Temperature variations affect gut microbiome diversity and structure in both ectothermic and endothermic animals. Here, we aimed to characterize temperature-induced changes in the gut microbiotas of adjacent natural populations of the least horseshoe bat (Rhinolophus pusillus) which hibernate at different ambient temperatures. We found that the ambient temperature significantly affected the α-diversity, but not the β-diversity, of the gut microbiota. Bats hibernating at cooler temperatures experienced more drastic shifts in gut microbiome structure, with consequent effects on energy-related metabolic pathways. Our results provide novel insights into the effects of ambient temperature on the gut microbiotas of hibernating animals.

## INTRODUCTION

The gut microbiota, as the most important symbionts of vertebrate hosts, influences nutrition, immunity, brain development, and fitness (1–4). The gut microbiota can even increase vertebrate phenotypic plasticity: alterations in gut microbiome composition may support the adaptation of the host to a changing environment (5). Microbes in the gut are affected by a variety of factors, including host phylogeny, dietary habits, age, and sex (6). Importantly, changes in ambient temperature (*T_a_*) strongly affect microorganism distributions worldwide. As global warming may lead to severe temperature fluctuations, it is interesting and important to investigate whether these temperature changes also affect gut microbial communities.

Studies of temperature acclimation in both ectothermic and endothermic animals have revealed that temperature changes affect gut microbial community composition. For example, exposure to a warmer *T_a_* (28°C) significantly increased the relative abundance of the phylum *Planctomycetes* and genus Mycobacterium in the gut microbiota of the tadpole (Lithobates pipiens) (7). Similarly, heat stress significantly decreased the relative abundance of *Firmicutes* and increased that of *Bacteroidetes* in the fecal microbiotas of laying hens (8), while 4 weeks of heat exposure increased the relative abundance of *Lactobacillus* and *Oscillospira* and decreased that of *Blautia* and *Allobaculum* in the gut microbiotas of rats (9). Finally, an increase in *T_a_* from 10°C to 20°C correspondingly decreased the microbial diversity in the gut of the red-backed salamander (Plethodon cinereus) (10), while a 2°C to 3°C increase in *T_a_* reduced the gut microbial diversity of the common lizard (Zootoca vivipara) by 34% (11). As the global mean surface temperature has increased year to year for the past 5 years (12), these previous studies suggest that global warming may have rapid effects on the compositions of the gut microbiotas of a variety of vertebrates.

Global warming may also affect the gut microbiotas of hibernators. Hibernator gut microbiotas are substantially remodeled by winter fasting (13–16) to favor those microbial taxa that degrade and utilize endogenous substrates produced by the host (such as mucin glycans) (17). The altered microbiome may modulate white adipose tissue (WAT) browning, as well as brown adipose tissue (BAT) activity (18, 19); BAT is responsible for nonshivering thermogenesis and provides extra heat for arousal from torpor (20). In the gut microbiotas of hibernating animals, urease activity is high, which helps them to recoup nitrogen from urea, thereby maintaining protein homeostasis in the body (21, 22). Thus, microbiome alterations in response to winter fasting play an essential role in the maintenance of homeostasis, and a gut microbiome with a stable composition and diversity is therefore critical for hibernators. As the body temperature (*T_b_*) of a hibernating mammal is typically close to the hibernacula temperature for effective hibernation (23), it is urgently necessary to investigate whether temperature fluctuations alter the gut microbial communities of hibernating animals.

**FIG 1:**
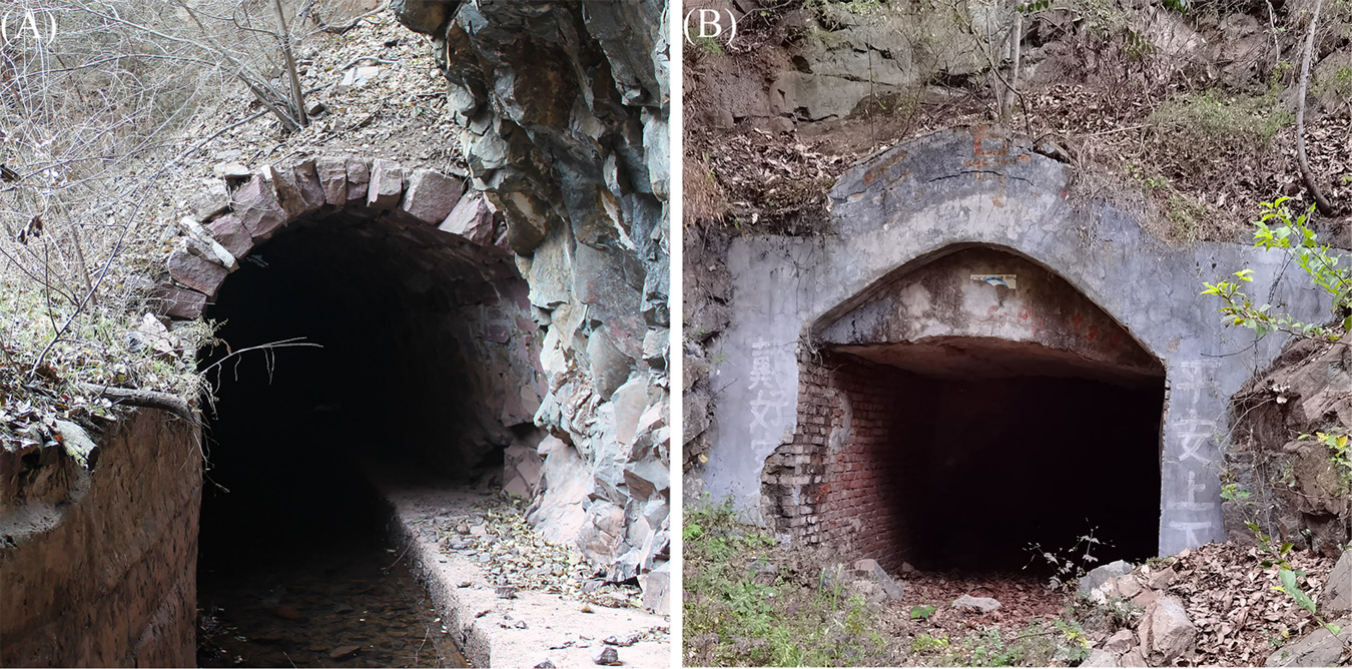
Images of the two sites included in this study: artificial canal tunnel (canal site) (A) and abandoned mine tunnel (mine site) (B).

In this study, we aimed to explore this knowledge gap using two populations of the least horseshoe bat (Rhinolophus pusillus) that inhabit adjacent caves (separated by a linear distance of about 2 km) in Jiyuan, China. One population inhabits an artificial canal tunnel, while the other inhabits an abandoned mine tunnel (Fig. 1). There is a seasonal river between the habitats, making it easier for the flying bats to cross. In summer, these distinct habitats have similar *T_a_*s. In winter, however, the canal site is cooler and the mine site is warmer (a difference of about 5°C). This system provides an opportunity to study the effects of ambient temperature on the gut microbiota of a hibernating animal under entirely natural conditions. We used 16S rRNA gene high-throughput sequencing to compare changes in gut microbial diversity and composition between the two populations at each stage. We predicted that the difference in hibernacula *T_a_* would (i) significantly impact the gut microbiome of hibernating *R. pusillus* bats and (ii) affect the metabolic pathways that support host adaptation to different microhabitat temperatures. Our results may provide insight into the role played by *T_a_* in altering gut microbiome composition and diversity during the extreme physiological conditions of hibernation.

## RESULTS

### Microbial community diversity.

Sequencing of the V3 to V4 region of the bacterial 16S rRNA gene yielded 2,463,336 high-quality reads from 23 bats, corresponding to approximately 107,102 sequences per sample (see Table S1 in the supplemental material). Rarefaction curves plateaued as the sequencing depth increased, indicating that the majority of the microbial diversity in our samples was covered by sequencing (Fig. S1).

**FIG 2:**
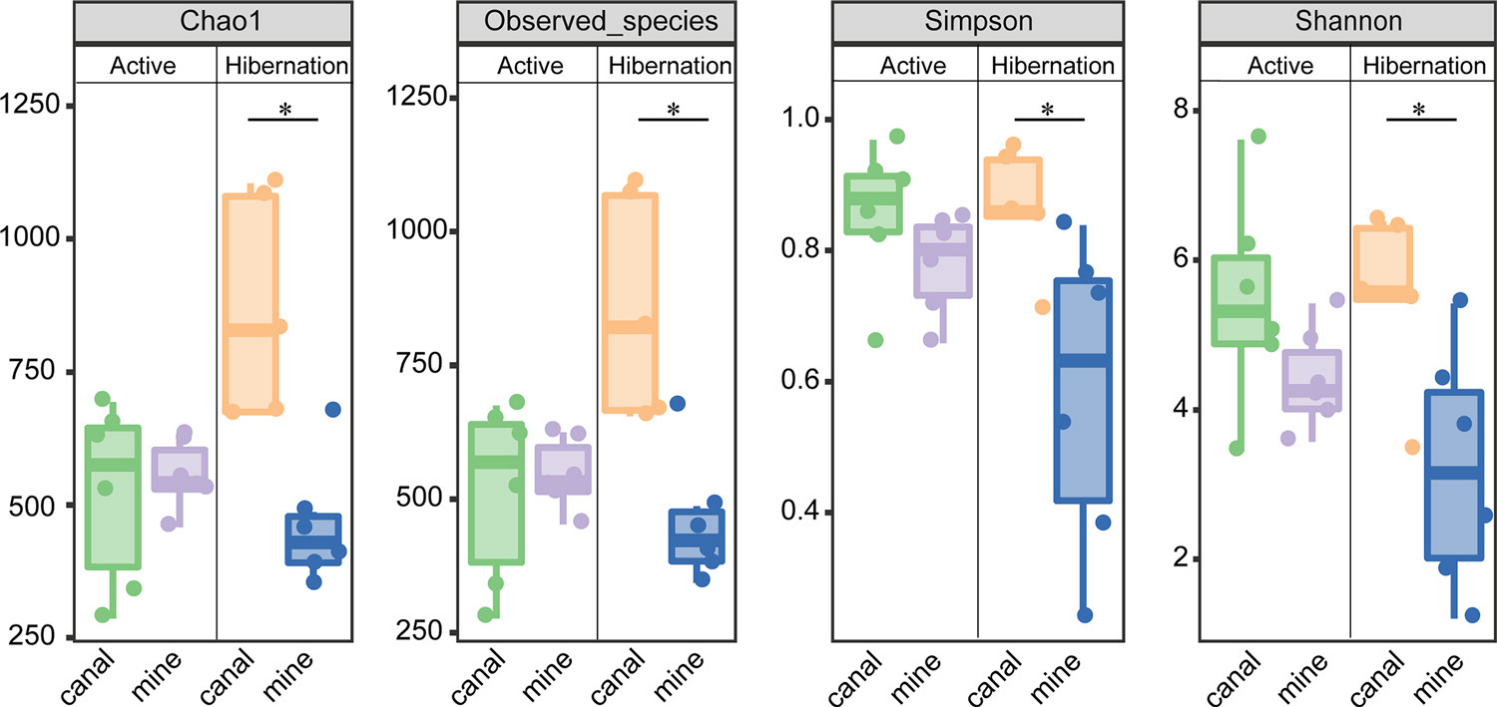
Indexes of α-diversity for the gut microbial communities of the four groups of bats. Significant differences between sites are indicated with asterisks (*, *P *< 0.05).

Indexes of α-diversity (Chao1, observed species, Simpson, and Shannon) were similar at the canal site and the mine site in summer (active groups; all indexes, *P > *0.05), but the α-diversity was significantly greater at the canal site than at the mine site in winter (hibernating groups; all indexes, *P *< 0.05) (Fig. 2). Neither body weight nor *T_b_* affected gut microbial diversity during the active period; however, *T_b_* had a significant effect on the α-diversity of the gut microbiome in the hibernating groups (Table S2).

**FIG 3:**
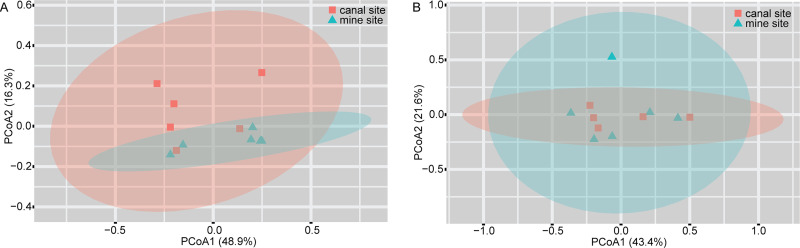
PCoA plots of microbial community structure based on weighted UniFrac distances during the active period (A) and hibernation (B).

Principal-coordinate analysis (PCoA) of the weighted UniFrac distances showed that the gut microbial structure (i.e., β-diversity) was similar at the two sites during the active period (permutational multivariate analyses of variance [PERMANOVA], *R*^2^ = 0.152; *P = *0.083) and during hibernation (PERMANOVA, *R*^2^ = 0.090; *P = *0.443) (Fig. 3). During the active period, the first two principal coordinates explained 35.2% of the total variance, while during hibernation, the first two principal coordinates explained 43.4% of the total variance. The dispersions in weighted UniFrac distances, as calculated using permutational analysis of multivariate dispersions (PERMDISP), were homogeneous between the sites both during the active period (*F *= 0.771; *P = *0.259) and during hibernation (*F *= 0.639; *P = *0.350).

### Divergence in microbial community composition between hibernating groups.

As this study aimed to determine the effects of *T_a_* on the gut microbiota during hibernation, we focused on differences in the composition of the gut microbial community between the two hibernating groups. The gut microbiotas of both groups were primarily comprised of six major bacterial phyla, with most sequences falling within *Proteobacteria* (53.9 ± 8.2%), followed by *Chlamydiae* (11.0 ± 7.8%), *Firmicutes* (10.7 ± 3.0%), *Actinobacteria* (8.1 ± 2.4%), *Tenericutes* (7.2 ± 6.8%), and *Bacteroidetes* (1.4 ± 0.4%). About 92.3% of all bacteria in both groups were assigned to these six phyla. However, three of these phyla (*Firmicutes*, *Actinobacteria*, and *Tenericutes*) were significantly differentially abundant in the hibernating bats between the two sites (Table S3). Similarly, the six most abundant bacterial genera, representing about 44% of the total bacteria, were identical in both hibernating groups. However, one genus (Staphylococcus) was significantly more abundant in the bats from the canal site than in the bats from the mine site.

**FIG 4:**
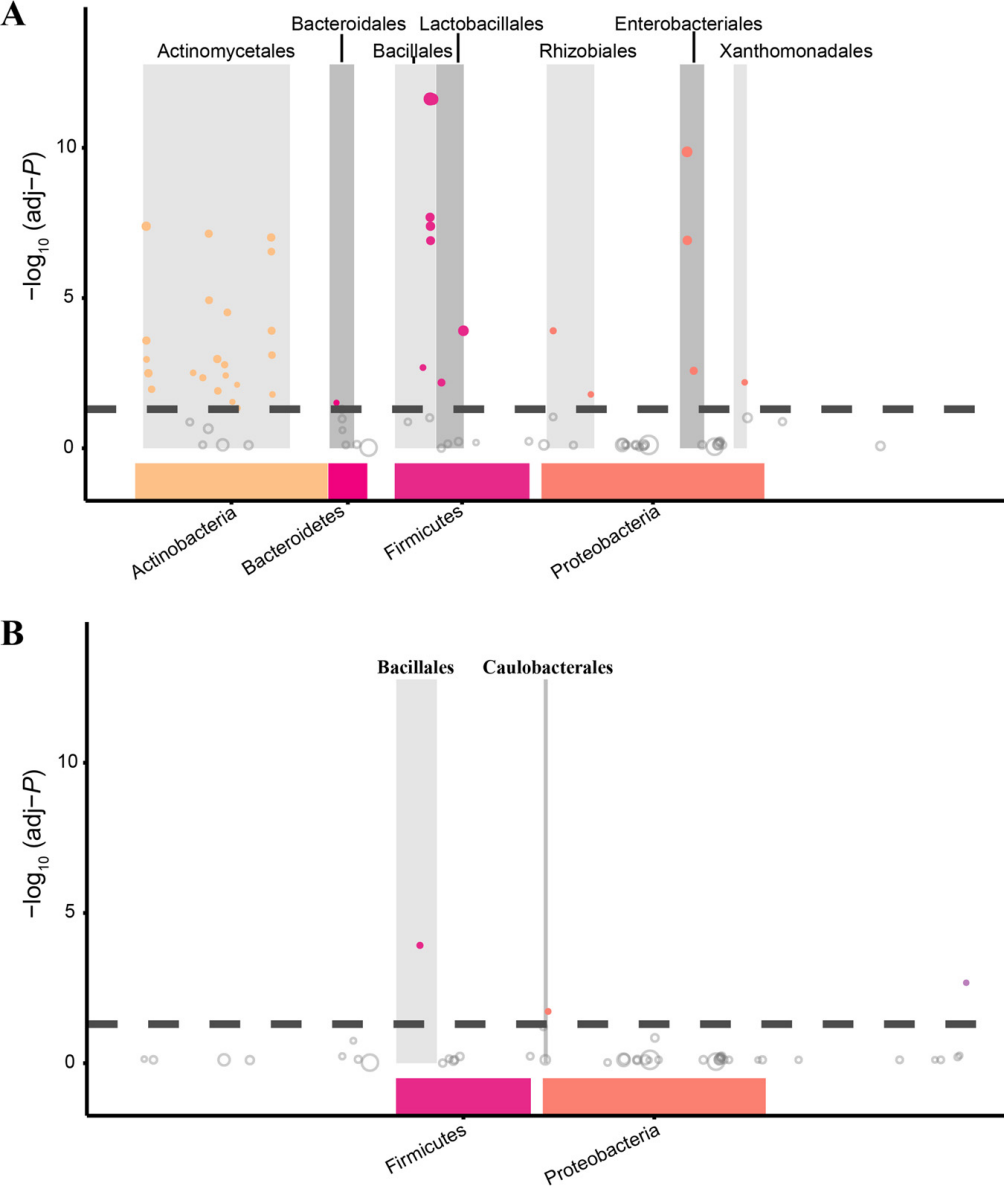
Manhattan plots showing ASVs that were enriched at the canal site compared to the mine site (A) and at the mine site compared to the canal site (B). Significantly differentially abundant ASVs are depicted as solid circles. The dashed line shows the significance threshold, which corresponds to a false-discovery rate (FDR)-corrected *P* value of 0.05. Dot colors indicate taxonomic affiliation.

We identified 5,353 amplicon sequence variants (ASVs) in the hibernating groups, and we classified them into 30 phyla, 83 classes, 183 orders, 337 families, and 611 genera. In total, 365 ASVs (6.82%) were shared between the two groups of hibernating bats. We identified 74 ASVs that were significantly differentially abundant between the hibernating groups (adjusted *P* value [adj-*P*], <0.05) (Table S4). Of these, most fell into the phyla *Actinobacteria* (28/74; 37.8%), *Proteobacteria* (20/74; 27.0%), and *Firmicutes* (12/74; 16.2%). More ASVs were differentially abundant at the canal site than at the mine site (Fig. 4). For 13 ASVs, the |log fold change in abundance (FC)| was greater than the arbitrary threshold (5), but only a single ASV (ASV 2734; phylum *Proteobacteria*, genus Pseudomonas) was significantly more abundant at the mine site than at the canal site (Table S4). The remaining 12 ASVs were significantly more abundant at the canal site than at the mine site; these ASVs fell into the phylum *Firmicutes*, genus Staphylococcus (ASV1, ASV2, ASV4, ASV5, and ASV10); the phylum *Actinobacteria*, genera *Brevibacterium* (ASV6), Mycobacterium (ASV7), and *unidentified_g_Pseudonocardiaceae* (ASV8 and ASV12); and the phylum *Proteobacteria*, genera *Gluconacetobacter* (ASV3 and ASV11) and Klebsiella (ASV9).

### Differences in microbial functions between hibernating groups.

PICRUSt was used to explore differences in microbiome function between the hibernating bats at the two sites. Four KEGG pathways were significantly enriched in the bats from the canal site: butirosin and neomycin biosynthesis; biosynthesis of 12-, 14-, and 16-membered macrolides; proteasome; and secondary bile acid biosynthesis (|logFC| > 1; all adj-*P*, <0.05) (Table 1).

**TABLE 1:** KEGG pathways significantly more abundant in the canal group than in the mine group

Pathway	logFC	SE	*P*	adj-*P*
Butirosin and neomycin biosynthesis	6.850	1.703	5.75 E−05	0.006
Biosynthesis of 12-,14-, and 16-membered macrolides	3.390	0.847	6.21 E−05	0.006
Proteasome	1.422	0.384	2.14 E−04	0.010
Secondary bile acid biosynthesis	1.047	0.283	2.17 E−04	0.010

## DISCUSSION

Heat acclimation experiments in both ectotherm and endotherm hosts have shown that variations in environmental temperature significantly affect the gut microbial community (9, 24–26). In this study, we used high-throughput 16S rRNA gene sequencing to investigate the impact of microhabitat temperature variations on gut microbial composition and diversity in *R. pusillus*. We compared free-living *R. pusillus* bats in two natural habitats rather than using captive bats in artificial habitats because captive feeding may increase the abundance of human-associated microorganisms in the gut, lowering the overall richness and diversity of the gut microbiota (27, 28).

Despite the impossibility of excluding all variations between the two populations with the exception of *T_a_*, this experiment was designed to minimize these variations. First, the two populations were located geographically close together (2 km apart), with a seasonal river between the habitats. This river simplifies movement between the two habitats. We observed interactions between bats from the different habitats during the active season; bats from the two habitats were distinguishable because both populations had been tagged with aluminum rings in previous investigations of chiropteran diversity in the same sampling areas (our unpublished data). These interactions may have mitigated the effects of population isolation and drift on gut microbial composition. Second, external climatic factors did not differ significantly between the two sites in summer or winter, and the α- and β-diversity indexes were similar in the gut microbiomes of the active groups (which had similar *T_a_*s). This suggests that the two bat populations were exposed to similar climatic regimes and likely had similar diets, thus reducing the effects of climate and dietary differences on the gut microbiotas of the hibernating bats. Third, we marked hibernating bats with aluminum rings to ensure that the sampled bats had hibernated at different temperatures for at least 30 days, and only adult males of similar body weights were sampled. These precautions minimized the confounding influence of individual physiological characters (e.g., age, sex, and weight). In addition, our analysis showed that microbial diversity in the gut was not significantly impacted by body weight.

The α-diversity of the gut microbiomes of the hibernating bats was significantly affected by differences in *T_b_*, induced by variations in *T_a_*. In winter, bats at the canal site had a significantly lower *T_b_* and significantly higher indexes of microbial α-diversity, which may reflect a maximization of microbial function. In contrast to the previous heat acclimation experiments, there were no significant differences in gut microbial structure during hibernation between the sites. However, relatively high temperatures are typically used in heat acclimation experiments, which may have detrimental effects on host weight and health, depressing fitness and immunological function (9). These negative physiological effects may influence gut microbial diversity and composition. At neither site in the natural system did the *T_a_* likely reach the physiological limit of *R. pusillus* for hibernation, as some bat species are known to hibernate in warm caves (20°C) (29). During hibernation, bats consume stored fat and do not feed, even during periods of interbout arousals (23). The similarity of the gut microbial β-diversity in the hibernating populations at the different sites with different *T_a_*s suggested that stabilization of the gut microbiota is important for hibernation.

Similar to some bat species (14, 30) and flying birds (30, 31), the predominant microbes in *R. pusillus* gut belonged to the phylum *Proteobacteria*. Even though most species of *Proteobacteria* are pathogenic (32), abundant volatile *Proteobacteria* may optimize microbial function and reduce the gut microbial biomass, decreasing the host weight and thus facilitating flight (33). Although the difference in ambient temperature during hibernation did not affect the relative abundance of *Proteobacteria*, the relative abundance of *Firmicutes* and *Actinobacteria* decreased sharply in bats from the mine site, while the relative abundance of the *Tenericutes* decreased in bats from the canal site. Consistent with this, a negative association between *Firmicutes* abundance and ambient temperature has previously been reported in ectothermic and ectothermic species (7, 11). For example, in Rhinolophus ferrumequinum, *Tenericutes* were less abundant at 12°C (*T_b_*) during the torpor stage than during the interbout arousal stage, when normothermic temperatures of around 30°C were sustained for short periods (14). However, the molecular mechanisms underlying this negative relationship remain unknown.

One ASV that was highly abundant at the mine site compared to the canal site fell into the genus Pseudomonas. Pseudomonas species are known to generate energy by breaking down lipids (34), and this energy may be used for basic metabolism and interbout arousals. Unexpectedly, many ASVs substantially more abundant at the canal site than at the mine site were classified into the genera Staphylococcus, Mycobacterium, and Klebsiella, which suggests that colder ambient temperatures during hibernation may increase pathogen invasion. Similarly, enhanced intestinal immune system function in hibernating ground squirrels (35) implies that colder environments may facilitate pathogen invasion. Further study is needed to determine whether potentially pathogenic bacteria were more abundant in the guts of hibernating bats at the canal site due to the colder ambient temperature of this site or due to a proliferation of pathogens at this site during the winter.

We hypothesized that the decreased ambient winter temperature at the canal site would enrich the energy metabolism pathways of bats there compared to those of bats at the mine site. Consistent with this hypothesis, three metabolism-related pathways were enriched in the canal bat guts compared to the mine bat guts. Hibernation temperature differences also affected the secondary bile acid biosynthesis pathway, which is associated with energy metabolism: Secondary bile acids, such as taurolytocholic acids and deoxycholic acid, contribute to BAT activity and brown WAT (18); BAT is the first source of arousal energy (20).

In conclusion, this study provides the first estimate of the relationship between ambient temperature and gut microbes in free-living hibernators during torpor. We found that differences in *T_a_* during hibernation led to significant changes in gut microbial α-diversity and associated energy metabolism pathways in the host hibernators. However, *T_a_* did not affect the β-diversity of the gut microbiota, emphasizing the importance of gut microbial stabilization to the bat hibernation process. Our findings also provide insights into the temperature-responsive mechanisms that influence animal fitness via the gut microbiome in wild-living populations.

## MATERIALS AND METHODS

### Habitat descriptions and sample collection.

The artificial canal tunnel (35.21°N, 112.13°E) and the abandoned mine tunnel (35.22°N, 112.15°E) are located in Jiyuan City, China. The two habitats are adjacent, separated by a linear distance of about 2 km. There is a seasonal river between the two habitats. The artificial canal tunnel (Fig. 1A) is about 800 m long, with two entrances. Water flows between the two entrances along the bottom of the tunnel at a depth of approximately 30 cm. Least horseshoe bats roost on the ceiling of the cave, about 170 to 180 m from the downstream entrance. The main cave of the mine tunnel (Fig. 1B) is about 150 m long, and the least horseshoe bats live in a cave (about 50 m long) that branches off near the end of the main cave. No water flows through the mine tunnel, nor is there any surface water.

From 1 January 2019 to 31 December 2019, the ambient temperature and ambient humidity were automatically recorded every 2 h, 24 h a day, in each location, using a USB temperature recorder (HE173; Huato Electric Co., Ltd., China). In both locations, *T_a_* was relatively constant throughout the year (11.6°C to 16.8°C in the canal tunnel; 17.1°C to 18.0°C in the mine tunnel).

In August (i.e., during the active period) and December (i.e., during the hibernation period) of 2019, six adult male bats per location were captured using mist nets or by hand. The bats were identified as adults based on the estimated degree of ossification of the epiphyseal spacing (36). By counting the bats tagged with aluminum rings, we determined which bats were hibernating at different temperatures. The body temperature of each collected bat was recorded using a handheld infrared laser thermometer (62 Max+; Fluke, USA).

The environmental data were collected 30 days before sampling (for the active groups, from 16 July to 14 August; for the hibernating groups, from 15 November to 14 December), as shown in Table 2. Climatic data, including the mean air temperature (MAT), mean precipitable water (MPW), mean relative humidity (MRH), and mean atmospheric pressure (MAP), were collected every 6 h using RNCEP with R 4.2.2 (37). The ambient temperature (*T_a_*) and ambient humidity of microhabitats were automatically recorded every 2 h in each location using a USB temperature recorder (HE173; Huato Electric Co., Ltd.). The significance of differences between sites was determined by analysis of variance (ANOVA).

**TABLE 2:** Bat habitat and weight characteristics during the active and hibernation periods^*a*^

Data type	Characteristic	Data collected in summer at:		Data collected in winter at:	
		Canal site	Mine site	Canal site	Mine site
External climate data^*b*^	MAT (°C)	24.1 ± 2.2a	24.1 ± 2.2a	0.7 ± 0.1b	0.7 ± 0.1b
	MPW (kg/m^2^)	41.0 ± 3.7a	41.1 ± 3.7a	7.9 ± 0.7b	7.9 ± 0.7b
	MRH (%)	87.9 ± 8.0a	87.9 ± 8.0a	55.6 ± 5.1b	55.6 ± 5.1b
	MAP (kPa)	93.1 ± 8.5a	93.1 ± 8.5a	94.8 ± 8.7b	94.8 ± 8.7b
Microhabitat data^*c*^	*T_a_* (°C)	16.2 ± 0.6a	17.9 ± 0.1a	11.9 ± 0.3b	17.1 ± 0.0a
Specimen data^*d*^	*T_b_* (°C)	31.2 ± 0.4a	31.9 ± 0.4a	11.7 ± 0.1b^*e*^	18.8 ± 0.3c
	Wt (g)	6.3 ± 0.1a	6.1 ± 0.1a	7.3 ± 0.3b^*e*^	7.0 ± 0.2b

a Values shown are means ± SE. Different letters (a, b, c) in the same row indicate significant differences (*P* < 0.05).

b *n* = 120.

c *n* = 360.

d *n* = 6.

e Means based on a sample size of 5.

The MAT, MPW, MRH, and MAP values were not significantly different between the sites in either summer or winter. There was no significant difference in *T_a_* between the artificial canal tunnel and the abandoned mine tunnel during the summer, but in winter, the mean *T_a_* in the mine tunnel was significantly greater than that in the canal tunnel (a difference of about 5°C; Table 2). The ambient humidity in both locations was over 95% at all time points measured.

Although the hibernating bats were significantly heavier than the active bats, there were no significant differences in body weight between the sites in either period (Table 2). The mean *T_b_* was similar between the bats at both sites during the active period, but during hibernation, the mean *T_b_* of the mine site bats was significantly higher than that of the canal site bats (Table 2). Specific body weights and *T_b_*s for each collected specimen are listed in Table S1 in the supplemental material.

Our preliminary experiments showed that it was difficult to obtain feces during winter fasting and that the microbial genomic DNA obtained via rectal swabs was insufficient for the construction of a sequencing library. Therefore, we sampled gut contents directly from the intestines: intact intestines were rapidly removed from the euthanized bats, and the gut contents were collected on ice. The gut contents were immediately frozen and stored in liquid nitrogen until sequencing. We successfully obtained and sequenced the gut contents of 23 bats; one sample collected in winter from the canal site was contaminated during transport and removed from the study. Animal experiments were conducted in accordance with the National Animal Research Authority of Northeast Normal University, China (approval number NENU-20080416).

### DNA extraction and 16S rRNA gene high-throughput sequencing.

The gut contents from each specimen were homogenized. Genomic DNA was extracted using a FastDNA Spin extraction kit (MP Biomedicals, Santa Ana, CA, USA). The quantity and quality of the extracted DNA were determined using a NanoDrop 2000 spectrophotometer (Thermo Fisher Scientific, Waltham, MA, USA) and 1.2% agarose gel electrophoresis.

The V3 to V4 region of the bacterial 16S rRNA gene was amplified using PCR with universal primers (338F, 5′-ACTCCTACGGGAGGCAGCA-3′, and 806R, 5′-GGACTACHVGGGTWTCTAAT-3′). Sample-specific barcodes were incorporated into the primers for multiplex sequencing. Each PCR mixture contained 2 μL genomic DNA template (20 ng/μL), 5 μL Q5 reaction buffer, 5 μL Q5 high-fidelity GC buffer (5×), 2 μL deoxynucleoside triphosphates (dNTPs; 2.5 mM each), 1 μL of each primer (10 μM), 0.25 μL Q5 high-fidelity DNA polymerase (5 U/μL), and 8.75 μL ddH_2_O. The PCR conditions were as follows: predenaturation at 98°C for 2 min; 30 cycles of 98°C for 15 s, 55°C for 30 s, and 72°C for 30 s; and a final extension at 72°C for 5 min.

The PCR amplicons were purified and quantified. A sequencing library was constructed, and high-throughput sequencing was performed on an Illumina NovaSeq PE250 (paired end, 250 bp) sequencing platform by the Shanghai Personal Biotechnology Co., Ltd. (Shanghai, China).

### Data processing.

The sequences were processed using the DADA2 pipeline in QIIME2 v2019.4 (38). In brief, sequences were assigned to samples based on the unique barcode. After removing primer sequences, the raw sequences were denoised and merged. Finally, chimeras were removed. The remaining high-quality sequences were considered amplicon sequence variants (ASVs). Singleton ASVs were removed. The QIIME feature table rarefy function was used at 95% of the minimum sequencing depth to minimize the differences in sequencing depths across samples. Species were taxonomically identified by subjecting the ASVs to a BLAST search against the SILVA database (release 132) (39).

### Statistical analysis.

Alpha diversity indexes (Chao1, observed species, Shannon, and Simpson) were calculated based on the ASVs to estimate the microbiome α-diversity of each bat group. ANOVAs and Mann-Whitney U tests were used to estimate the significance of differences among groups. We also used simple linear regressions to test whether body weight or *T_b_* affected the alpha diversity based on these metrics. Structural variations in microbial communities across samples were explored using principal-coordinate analyses (PCoAs) based on weighted UniFrac distances. Permutational analysis of multivariate dispersions (PERMDISP) with 999 permutations was performed to determine the homogeneity of the dispersions in the weighted UniFrac distance matrix. Significant differences in microbiota structure among groups were assessed using permutational multivariate analyses of variance (PERMANOVAs) with 999 permutations in R v4.2.2 (40).

Using the metagenomeSeq package in R v4.2.2 (40), we fitted each ASV distribution using a zero-inflated log-normal model and computed the significance of the differences in abundance among groups. Manhattan plots were used to visualize the differentially abundant ASVs. PICRUSt2 (Phylogenetic Investigation of Communities by Reconstruction of Unobserved States) (41) was used to predict the KEGG metabolic pathways associated with the ASVs (42).

### Data availability.

The bacterial 16S rRNA gene sequences obtained in this study have been submitted to the National Center for Biotechnology Information (NCBI) Sequence Read Archive under accession number PRJNA923401 (summer canal site, samples F1901I to F1906I; summer mine site, samples F1907I to F1912I; winter canal site, samples F1913I to F1917I; winter mine site, samples F1919I to F1924I).
