# Supplementary material for: Effects of Microhabitat Temperature Variations on the Gut Microbiotas of Free-Living Hibernating Animals
Source: Microbiol Spectr. 2023 Jun 28;11(4):e00433-23. doi: 10.1128/spectrum.00433-23 (PMC10434193; doi:10.1128/spectrum.00433-23)
Supplement: Supplemental file 1 — Table S1. Download spectrum.00433-23-s0001.docx, DOCX file, 0.02 MB [file spectrum.00433-23-s0001.docx]

**TABLE S1** Individual data for each least horseshoe bat collected, including physical characteristics, number of sequences per sample, and alpha diversity indexes for the gut microbiome.

| **Sample** | | | **Ring number** | **Weight (g)** | ***T_b_* (°C)** | **Sequences number** | **Chao1** | **Observed**  **species** | **Shannon**  **diversity** | **Simpson**  **diversity** |
| --- | --- | --- | --- | --- | --- | --- | --- | --- | --- | --- |
| Active groups | canal site | F1901I | - | 6.3 | 32.5 | 98,958 | 651.5 | 647.0 | 6.174 | 0.904 |
|  |  | F1902I | - | 5.8 | 31.5 | 102,172 | 623.4 | 617.1 | 7.614 | 0.969 |
|  |  | F1903I | - | 6.3 | 30.9 | 83,307 | 336.5 | 335.8 | 5.597 | 0.917 |
|  |  | F1904I | - | 6.4 | 30.1 | 120,453 | 282.7 | 276.7 | 3.453 | 0.659 |
|  |  | F1905I | - | 6.3 | 31.9 | 102,406 | 524.4 | 519.7 | 4.825 | 0.819 |
|  |  | F1906I | - | 6.4 | 30.1 | 137,987 | 694.1 | 674.3 | 5.028 | 0.855 |
|  | mine site | F1907I | - | 6.4 | 29.7 | 102,028 | 535.6 | 527.0 | 4.322 | 0.849 |
|  |  | F1908I | - | 5.9 | 32.6 | 110,859 | 549.5 | 538.6 | 3.572 | 0.658 |
|  |  | F1909I | - | 5.7 | 32.2 | 99,435 | 619.2 | 615.3 | 4.908 | 0.841 |
|  |  | F1910I | - | 6.1 | 32.5 | 117,282 | 526.5 | 512.2 | 4.180 | 0.780 |
|  |  | F1911I | - | 6.2 | 31.9 | 115,885 | 632.8 | 626.2 | 5.417 | 0.821 |
|  |  | F1912I | - | 6.4 | 32.6 | 121,258 | 458.6 | 453.4 | 3.956 | 0.715 |
| Hibernating groups | canal site | F1913I | A00699 | 8.1 | 12.0 | 91,805 | 670.3 | 667.3 | 5.572 | 0.852 |
|  |  | F1914I | A00671 | 7.6 | 11.6 | 120,057 | 678.1 | 659.1 | 3.456 | 0.708 |
|  |  | F1915I | A00674 | 6.3 | 11.6 | 113,505 | 1101.0 | 1089.2 | 5.472 | 0.859 |
|  |  | F1916I | A00675 | 7.0 | 11.9 | 128,862 | 1080.8 | 1067.0 | 6.421 | 0.938 |
|  |  | F1917I | A00676 | 7.4 | 11.5 | 98,113 | 827.1 | 820.0 | 6.518 | 0.957 |
|  | mine site | F1919I | A00677 | 7.6 | 17.8 | 90,966 | 674.0 | 672.0 | 5.417 | 0.839 |
|  |  | F1920I | A00678 | 7.1 | 19.9 | 77,669 | 487.1 | 486.9 | 3.767 | 0.761 |
|  |  | F1921I | A00679 | 6.7 | 19.3 | 113,643 | 449.6 | 444.0 | 4.386 | 0.730 |
|  |  | F1922I | A00680 | 7.4 | 19.6 | 100,273 | 347.1 | 342.7 | 1.839 | 0.379 |
|  |  | F1923I | A00682 | 6.6 | 17.9 | 114,765 | 387.2 | 378.4 | 1.205 | 0.238 |
|  |  | F1924I | A00683 | 6.3 | 18.3 | 101,648 | 405.6 | 400.7 | 2.545 | 0.534 |
