# Supplementary material for: Effects of Microhabitat Temperature Variations on the Gut Microbiotas of Free-Living Hibernating Animals
Source: Microbiol Spectr. 2023 Jun 28;11(4):e00433-23. doi: 10.1128/spectrum.00433-23 (PMC10434193; doi:10.1128/spectrum.00433-23)
Supplement: Supplemental file 2 — Table S2. Download spectrum.00433-23-s0002.docx, DOCX file, 0.02 MB [file spectrum.00433-23-s0002.docx]

**TABLE S2** Simple linear regressions showing relationships between physiological characteristics (*T_b_* and body weight) and microbial diversity indices.

| **Groups** | **Model: predictor-response** | **Estimate ± SE** | ***R*^2^** | ***t*** | ***P*** |
| --- | --- | --- | --- | --- | --- |
| Active groups | *T_b_* -Chao1 | 0.002 ± 0.003 | -0.027 | 0.842 | 0.419 |
|  | *T_b_* -Simpson | -1.578 ± 3.434 | -0.077 | -0.459 | 0.656 |
|  | *T_b_* -Shannon | 0.045 ± 0.288 | -0.097 | 0.158 | 0.878 |
|  | *T_b_* -Observed_species | 0.002 ± 0.003 | -0.020 | 0.887 | 0.396 |
|  | Weight-Chao1 | 0.001 ± 0.001 | 0.040 | -1.208 | 0.255 |
|  | Weight-Simpson | -0.386 ± 0.803 | -0.075 | -0.481 | 0.641 |
|  | Weight-Shannon | 0.065 ± 0.064 | 0.002 | -1.008 | 0.337 |
|  | Weight-Observed_species | 0.001 ± 0.001 | 0.048 | -1.247 | 0.241 |
| Hibernating groups | *T_b_* -Chao1 | -0.012 ± 0.003 | 0.631 | -4.256 | **0.002** |
|  | *T_b_* -Simpson | -9.778 ± 3.943 | 0.294 | -2.274 | **0.049** |
|  | *T_b_* -Shannon | -1.322 ± 0.525 | 0.348 | -2.516 | **0.033** |
|  | *T_b_* -Observed_species | -0.012 ± 0.003 | 0.622 | -4.177 | **0.002** |
|  | Weight-Chao1 | 0.000 ± 0.000 | -0.111 | 0.023 | 0.982 |
|  | Weight-Simpson | 0.756 ± 0.796 | -0.009 | 0.950 | 0.367 |
|  | Weight-Shannon | 0.093 ± 0.102 | -0.016 | 0.917 | 0.383 |
|  | Weight-Observed_species | 0.000 ± 0.000 | -0.111 | 0.032 | 0.975 |

Significant results are in bold (*P* < 0.05).
