# Supplementary material for: Effects of Microhabitat Temperature Variations on the Gut Microbiotas of Free-Living Hibernating Animals
Source: Microbiol Spectr. 2023 Jun 28;11(4):e00433-23. doi: 10.1128/spectrum.00433-23 (PMC10434193; doi:10.1128/spectrum.00433-23)
Supplement: Supplemental file 3 — Table S3. Download spectrum.00433-23-s0003.docx, DOCX file, 0.02 MB [file spectrum.00433-23-s0003.docx]

**TABLE S3** Significant differences in the relative abundances of gut microbes at the phylum and genus level between the two hibernating groups. Values shown are means ± SE.

| **Taxon** | **canal site (%)** | **mine site (%)** | **Z** | ***P*** |
| --- | --- | --- | --- | --- |
| Phylum |  |  |  |  |
| Proteobacteria | 51.332 ± 8.015 | 56.067 ± 13.507 | -0.548 | 0.662 |
| Firmicutes | 17.841 ± 4.753 | 4.717 ± 1.258 | -2.739 | **0.004** |
| Chlamydiae | 6.733 ± 5.992 | 14.560 ± 13.270 | -0.366 | 0.792 |
| Actinobacteria | 14.937 ± 3.341 | 2.406 ± 0.497 | -2.739 | **0.004** |
| Tenericutes | 0.020 ± 0.015 | 13.180 ± 11.907 | -2.395 | **0.017** |
| Bacteroidetes | 1.421 ± 0.636 | 1.393 ± 0.569 | 0.000 | 1.000 |
| Genus |  |  |  |  |
| *Cupriavidus* | 18.177 ± 4.489 | 23.088 ± 7.061 | 0.000 | 1.000 |
| *Mycoplasma* | 0.003 ± 0.002 | 13.098 ± 11.922 | -1.057 | 0.429 |
| *Staphylococcus* | 11.967 ± 5.544 | 0.035 ± 0.015 | -2.745 | **0.004** |
| *Klebsiella* | 11.654 ± 10.357 | 0.000 ± 0.000 | -2.090 | 0.126 |
| *Sphingomonas* | 3.095 ± 0.953 | 3.896 ± 1.183 | -0.365 | 0.792 |
| *Rhodococcus* | 2.160 ± 0.294 | 1.609 ± 0.304 | -0.730 | 0.537 |
