# Supplementary material for: Effects of Microhabitat Temperature Variations on the Gut Microbiotas of Free-Living Hibernating Animals
Source: Microbiol Spectr. 2023 Jun 28;11(4):e00433-23. doi: 10.1128/spectrum.00433-23 (PMC10434193; doi:10.1128/spectrum.00433-23)
Supplement: Supplemental file 4 — Table S4. Download spectrum.00433-23-s0004.docx, DOCX file, 0.03 MB [file spectrum.00433-23-s0004.docx]

**TABLE S4** The ASVs significantly more abundant during winter.

| ID used only in this analysis | ASV id used only in this analysis | phylum | class | order | family | genus | logFC | *P* | adj-*P* |
| --- | --- | --- | --- | --- | --- | --- | --- | --- | --- |
| b0435c64d7881bb275f28258cc9b1d35 | ASV1 | Firmicutes | Bacilli | Bacillales | Staphylococcaceae | *Staphylococcus* | 7.283 | 1.55E-15 | 2.43E-12 |
| d20b79153ed4aa51868f58aed19ffc6b | ASV2 | Firmicutes | Bacilli | Bacillales | Staphylococcaceae | *Staphylococcus* | 7.265 | 1.78E-15 | 2.43E-12 |
| c662cb231b6389676feeed06ebedefbd | ASV3 | Proteobacteria | Gammaproteobacteria | Enterobacteriales | Enterobacteriaceae | *Gluconacetobacter* | 6.747 | 1.52E-13 | 1.39E-10 |
| 41f20f0fb4adc82865727663dd803e02 | ASV4 | Firmicutes | Bacilli | Bacillales | Staphylococcaceae | *Staphylococcus* | 6.069 | 3.08E-11 | 2.11E-08 |
| e5229654d9ad27ae71fbfcf6eab4559b | ASV5 | Firmicutes | Bacilli | Bacillales | Staphylococcaceae | *Staphylococcus* | 5.928 | 8.67E-11 | 4.10E-08 |
| 1f78e41d5572c3d312b5478b9fa1cb56 | ASV6 | Actinobacteria | Actinobacteria | Actinomycetales | Brevibacteriaceae | *Brevibacterium* | 5.923 | 9.01E-11 | 4.10E-08 |
| 0c617247006c359de0b5f8dc3c15049c | ASV7 | Actinobacteria | Actinobacteria | Actinomycetales | Mycobacteriaceae | *Mycobacterium* | 5.821 | 1.88E-10 | 7.32E-08 |
| c671a69d4bc4e084e2718c309747ed63 | ASV8 | Actinobacteria | Actinobacteria | Actinomycetales | Pseudonocardiaceae | *unidentified_Pseudonocardiaceae* | 5.763 | 2.82E-10 | 9.65E-08 |
| 7882fae92361e7fab5b115d5c372f422 | ASV9 | Proteobacteria | Gammaproteobacteria | Enterobacteriales | Enterobacteriaceae | *Klebsiella* | 5.719 | 3.85E-10 | 1.17E-07 |
| 45f399ce58ae1bc953dfd6a7e5d0ffd6 | ASV10 | Firmicutes | Bacilli | Bacillales | Staphylococcaceae | *Staphylococcus* | 5.686 | 4.85E-10 | 1.24E-07 |
| e40f9a95690813a4b99788bf180ba686 | ASV11 | Proteobacteria | Gammaproteobacteria | Enterobacteriales | Enterobacteriaceae | *Gluconacetobacter* | 5.682 | 4.98E-10 | 1.24E-07 |
| 943b95bc0ab3dcffc329e307a7b8d8d0 | ASV12 | Actinobacteria | Actinobacteria | Actinomycetales | Pseudonocardiaceae | *unidentified_Pseudonocardiaceae* | 5.548 | 1.26E-09 | 2.86E-07 |
| 94e2eac30c718f7a698710db82b9b4a1 | ASV13 | Actinobacteria | Actinobacteria | Actinomycetales | Mycobacteriaceae | *Mycobacterium* | 4.947 | 6.15E-08 | 1.20E-05 |
| adc51a6daf8fca43aa11ee8499c0efea | ASV14 | Actinobacteria | Actinobacteria | Actinomycetales | Nocardiaceae | *unidentified_Nocardiaceae* | 4.775 | 1.73E-07 | 3.07E-05 |
| ef4516f44aeb9d65dac7609fe335df74 | ASV15 | Actinobacteria | Actinobacteria | Actinomycetales | Pseudonocardiaceae | *unidentified_Pseudonocardiaceae* | 4.492 | 8.77E-07 | 0.000124 |
| ed5bc7b6b8181686ad5bd7c0db099bd3 | ASV16 | Firmicutes | Bacilli | Lactobacillales | Streptococcaceae | *Lactococcus* | 4.487 | 9.05E-07 | 0.000124 |
| 70ae63079b736bf3a1bc2b72d1089dac | ASV17 | Proteobacteria | Alphaproteobacteria | Rhizobiales | Bradyrhizobiaceae | *Bradyrhizobium* | 4.468 | 1.00E-06 | 0.000125 |
| 74bc50dc6bb482e339c8f68b5b5fea7c | ASV18 | Actinobacteria | Actinobacteria | Actinomycetales | Brevibacteriaceae | *Brevibacterium* | 4.322 | 2.24E-06 | 0.000266 |
| 0c97e760b1a868cfe839cb55863b10ac | ASV19 | Actinobacteria | Actinobacteria | Actinomycetales | Pseudonocardiaceae | *unidentified_Pseudonocardiaceae* | 4.104 | 7.07E-06 | 0.000805 |
| ac98c2e11523a83e59b97b1be4aa9b9d | ASV20 | Actinobacteria | Actinobacteria | Actinomycetales | Nocardiaceae | *Rhodococcus* | 4.035 | 1.00E-05 | 0.001095 |
| eedd9f11d12e84f9f4bf3ac708e998c9 | ASV21 | Actinobacteria | Actinobacteria | Actinomycetales | Brevibacteriaceae | *Brevibacterium* | 4.021 | 1.08E-05 | 0.001131 |
| df1b475992f9e9a81323e4863e047f7a | ASV22 | Actinobacteria | Actinobacteria | Actinomycetales | Nocardiaceae | *Smaragdicoccus* | 3.928 | 1.71E-05 | 0.001703 |
| 9c12774266de334d16afa8044e41c4e6 | ASV23 | Firmicutes | Bacilli | Bacillales | Planococcaceae | *Sporosarcina* | 3.876 | 2.21E-05 | 0.002082 |
| f7e813bf95ea7e2d46fb31a1a8921b38 | ASV24 | Actinobacteria | Actinobacteria | Actinomycetales | Nocardiaceae | *Rhodococcus* | 3.804 | 3.12E-05 | 0.002511 |
| 3c529c9ea59c53cdab1dcf07f476239d | ASV25 | Proteobacteria | Gammaproteobacteria | Enterobacteriales | Enterobacteriaceae | *Serratia* | 3.788 | 3.38E-05 | 0.002636 |
| d9f481ccc5ff931ee817259d41f5e2d2 | ASV26 | Actinobacteria | Actinobacteria | Actinomycetales | Micrococcaceae | *Arthrobacter* | 3.745 | 4.16E-05 | 0.003156 |
| 64a080e36662eed87d010b7c794b6fc3 | ASV27 | Actinobacteria | Actinobacteria | Actinomycetales | Brevibacteriaceae | *Brevibacterium* | 3.725 | 4.55E-05 | 0.003279 |
| d5e6d3b0c3c938796400892e89486b9d | ASV28 | Actinobacteria | Actinobacteria | Actinomycetales | Nocardiaceae | *unclassified_Nocardiaceae* | 3.689 | 5.40E-05 | 0.003783 |
| ba906141c4b064c46ef3de06098e3f64 | ASV29 | Proteobacteria | Gammaproteobacteria | Enterobacteriales | Enterobacteriaceae | *Klebsiella* | 3.676 | 5.72E-05 | 0.003908 |
| 64706b3d34171ae6a4928d980648b541 | ASV30 | Actinobacteria | Actinobacteria | Actinomycetales | Mycobacteriaceae | *Mycobacterium* | 3.641 | 6.73E-05 | 0.004489 |
| 8986e4b1f2111681f9591a4aaf5d9485 | ASV31 | Proteobacteria | Gammaproteobacteria | Enterobacteriales | Enterobacteriaceae | *Brenneria* | 3.554 | 1.00E-04 | 0.006361 |
| e0fe0a9365375ef1dad8f760dedaf0d3 | ASV32 | Proteobacteria | Gammaproteobacteria | Xanthomonadales | Xanthomonadaceae | *unidentified_Xanthomonadaceae* | 3.544 | 0.000105 | 0.006499 |
| 7faa2a31ae94f0502faf387d46e4bb18 | ASV33 | Firmicutes | Bacilli | Lactobacillales | Enterococcaceae | *Enterococcus* | 3.538 | 0.000108 | 0.006532 |
| 4fb385dc8ebc313a05f7c00de9dcff16 | ASV34 | TM7 | TM7-3 | unidentified_TM7-3 | unidentified_TM7-3 | *unidentified_TM7-3* | 3.525 | 0.000114 | 0.006776 |
| 6ce24280af6bdfb240af6d7421580ba1 | ASV35 | Actinobacteria | Actinobacteria | Actinomycetales | Nocardioidaceae | *unidentified_Nocardioidaceae* | 3.482 | 0.000138 | 0.007865 |
| c7f6482b954f7194d7f6676418e270a2 | ASV36 | TM7 | TM7-3 | EW055 | unidentified_EW055 | *unidentified_EW055* | 3.425 | 0.000177 | 0.009696 |
| ea29c1b3b4f8b01982feed38c82242ff | ASV37 | Actinobacteria | Actinobacteria | Actinomycetales | Corynebacteriaceae | *Corynebacterium* | 3.385 | 0.000211 | 0.01109 |
| a7a97259ab5b0fbb09f206a5db4e685a | ASV38 | Actinobacteria | Actinobacteria | Actinomycetales | Nocardiaceae | *Rhodococcus* | 3.353 | 0.000243 | 0.0123 |
| 20b1701e02f9b0d3f92c8f441c276045 | ASV39 | Actinobacteria | Actinobacteria | Actinomycetales | Pseudonocardiaceae | *unidentified_Pseudonocardiaceae* | 3.347 | 0.000249 | 0.01237 |
| a7adc7e48063d57592d9a6859ab1d10a | ASV40 | Proteobacteria | Gammaproteobacteria | Enterobacteriales | Enterobacteriaceae | *Serratia* | 3.282 | 0.000327 | 0.0157 |
| 77eb9e9cc6e5244fca5f65bc8c0ae89e | ASV41 | Actinobacteria | Actinobacteria | Actinomycetales | Pseudonocardiaceae | *unidentified_Pseudonocardiaceae* | 3.268 | 0.000348 | 0.01638 |
| 4562ff1c050e3384ef00cc255923f0c2 | ASV42 | Proteobacteria | Alphaproteobacteria | Rhizobiales | unidentified_Rhizobiales | *unidentified_Rhizobiales* | 3.261 | 0.000357 | 0.01655 |
| 56c63dc2d9b43bcbabd51efb0842f5d5 | ASV43 | Actinobacteria | Actinobacteria | Actinomycetales | Pseudonocardiaceae | *Pseudonocardia* | 3.173 | 0.000514 | 0.02266 |
| 775decdec0692b99858380123cf0939a | ASV44 | Firmicutes | Bacilli | Bacillales | Bacillaceae | *Bacillus* | 3.115 | 0.000651 | 0.02781 |
| be70201121f7d94a55e900b74693f4c2 | ASV45 | Actinobacteria | Actinobacteria | Actinomycetales | Nocardioidaceae | *Kribbella* | 3.1 | 0.00069 | 0.029 |
| 6e4a5b547a6809dcb56f8db9d83cfdc1 | ASV46 | Actinobacteria | Actinobacteria | Actinomycetales | Pseudonocardiaceae | *unidentified_Pseudonocardiaceae* | 3.094 | 0.000707 | 0.0293 |
| c2fc9004b57b6c6406e8a0f8ffacce4d | ASV47 | Actinobacteria | Actinobacteria | Actinomycetales | Nocardiaceae | *Smaragdicoccus* | 3.086 | 0.00073 | 0.0298 |
| 51741d6336487b6e2c91cb7ab5933366 | ASV48 | Bacteroidetes | Bacteroidia | Bacteroidales | Porphyromonadaceae | *Parabacteroides* | 3.071 | 0.000775 | 0.03069 |
| e56a43f578e05406f5a97539dd5c0cfa | ASV49 | Actinobacteria | Actinobacteria | Actinomycetales | Pseudonocardiaceae | *Pseudonocardia* | 3.01 | 0.000986 | 0.03743 |
| f0a94495609426ce4cd8c39413037cb0 | ASV50 | Actinobacteria | Actinobacteria | Actinomycetales | Nocardioidaceae | *unidentified_Nocardioidaceae* | 2.948 | 0.001252 | 0.04627 |
| 95484bbacc8c71775d52607d8bd3ed46 | ASV2709 | unclassified_Bacteria | unclassified_Bacteria | unclassified_Bacteria | unclassified_Bacteria | *unclassified_Bacteria* | -2.963 | 0.001181 | 0.04421 |
| f355666853890535f8e8e531a0ad732e | ASV2710 | Firmicutes | Clostridia | Clostridiales | Clostridiaceae | *unidentified_Clostridiaceae* | -3.034 | 0.000899 | 0.03461 |
| 535d0fc80bfaf28553633708ea84517d | ASV2711 | Proteobacteria | Gammaproteobacteria | Pseudomonadales | Pseudomonadaceae | *Pseudomonas* | -3.067 | 0.000788 | 0.03077 |
| 26115f0250c167bccbc8641c83970d23 | ASV2713 | unclassified_Bacteria | unclassified_Bacteria | unclassified_Bacteria | unclassified_Bacteria | *unclassified_Bacteria* | -3.08 | 0.000749 | 0.03011 |
| 78e3f0e47e713c6386d159d304d56ed3 | ASV2714 | Bacteroidetes | Bacteroidia | Bacteroidales | Porphyromonadaceae | *unidentified_Porphyromonadaceae* | -3.15 | 0.000565 | 0.0245 |
| 05a1380a79c6d357617ddd17ad496515 | ASV2716 | Proteobacteria | Alphaproteobacteria | Caulobacterales | Caulobacteraceae | *unclassified_Caulobacteraceae* | -3.216 | 0.000431 | 0.0193 |
| 6337a85e25b650d84d01a90ee2b5f1fd | ASV2717 | Bacteroidetes | Bacteroidia | Bacteroidales | Marinilabiaceae | *unidentified_Marinilabiaceae* | -3.243 | 0.000385 | 0.01754 |
| 6bb18169a412151ea7e14094514eb2ef | ASV2718 | Bacteroidetes | Bacteroidia | Bacteroidales | Porphyromonadaceae | *unidentified_Porphyromonadaceae* | -3.291 | 0.000316 | 0.01543 |
| bad527bd07bd6d63039cf63a729349c2 | ASV2719 | Firmicutes | Bacilli | Bacillales | Planococcaceae | *Planococcus* | -3.361 | 0.000235 | 0.0121 |
| 2fad32e3689865d56dea071690c9864e | ASV2720 | Bacteroidetes | Flavobacteriia | Flavobacteriales | Flavobacteriaceae | *unidentified_Flavobacteriaceae* | -3.39 | 0.000206 | 0.01106 |
| 1c2fea60700f7598a50f083fb50845df | ASV2721 | Bacteroidetes | Bacteroidia | Bacteroidales | SB-1 | *unidentified_SB-1* | -3.457 | 0.000154 | 0.008613 |
| 80b6d13ab02afdb4aae62b4f148fdfbc | ASV2722 | Bacteroidetes | Flavobacteriia | Flavobacteriales | Flavobacteriaceae | *unidentified_Flavobacteriaceae* | -3.491 | 0.000133 | 0.007728 |
| 9fc0b28383306cf2dfa42c129ce19d86 | ASV2723 | Proteobacteria | Gammaproteobacteria | Pseudomonadales | Pseudomonadaceae | *Pseudomonas* | -3.56 | 9.75E-05 | 0.006346 |
| f8eba894ab3d919af141f74bedb5caf2 | ASV2724 | Bacteroidetes | Bacteroidia | Bacteroidales | Marinilabiaceae | *unidentified_Marinilabiaceae* | -3.725 | 4.56E-05 | 0.003279 |
| 2a53222dccdd5259cb0554117ee0ce7b | ASV2725 | Proteobacteria | Gammaproteobacteria | Pseudomonadales | Pseudomonadaceae | *unclassified_Pseudomonadaceae* | -3.814 | 2.98E-05 | 0.002467 |
| 55cfc619b56dbe98c76b2f9b1cd8f8b7 | ASV2726 | Proteobacteria | Gammaproteobacteria | Pseudomonadales | Pseudomonadaceae | *Pseudomonas* | -3.83 | 2.77E-05 | 0.002364 |
| 148e9e2d1a8f4c358bbb7e04a4ec3c12 | ASV2727 | Proteobacteria | Gammaproteobacteria | Alteromonadales | Alteromonadaceae | *Marinobacter* | -3.854 | 2.45E-05 | 0.002164 |
| b4afa71b078c97a5a83cf32926cd8de6 | ASV2728 | unclassified_Bacteria | unclassified_Bacteria | unclassified_Bacteria | unclassified_Bacteria | *unclassified_Bacteria* | -3.865 | 2.34E-05 | 0.002129 |
| d165f7a88657a633a3793a69ccacf96c | ASV2729 | Proteobacteria | Gammaproteobacteria | Pseudomonadales | Pseudomonadaceae | *Pseudomonas* | -3.924 | 1.75E-05 | 0.001703 |
| a2cadbb65f5838e876743c64d8060025 | ASV2730 | Chloroflexi | Anaerolineae | SBR1031 | SHA-31 | *unidentified_SHA-31* | -4.471 | 9.89E-07 | 0.000125 |
| 012752de1c07c17dbdb29a278e1d85b1 | ASV2731 | Firmicutes | Bacilli | Bacillales | Planococcaceae | *Planococcus* | -4.512 | 7.88E-07 | 0.00012 |
| ec65d133e678ab9848f27162c6ef6235 | ASV2732 | Proteobacteria | Gammaproteobacteria | Pseudomonadales | Pseudomonadaceae | *Pseudomonas* | -4.675 | 3.10E-07 | 4.98E-05 |
| 51e0b2001a13276e7cb7c8521cf56d97 | ASV2733 | Proteobacteria | Gammaproteobacteria | Alteromonadales | Alteromonadaceae | *Cellvibrio* | -4.768 | 1.80E-07 | 3.07E-05 |
| 508b8a2dfb00bd92b2c230a49b5439bc | ASV2734 | Proteobacteria | Gammaproteobacteria | Pseudomonadales | Pseudomonadaceae | *Pseudomonas* | -5.073 | 2.82E-08 | 5.92E-06 |
