# Supplementary figures and images for: Effects of Microhabitat Temperature Variations on the Gut Microbiotas of Free-Living Hibernating Animals
Source: Microbiol Spectr. 2023 Jun 28;11(4):e00433-23. doi: 10.1128/spectrum.00433-23 (PMC10434193; doi:10.1128/spectrum.00433-23)

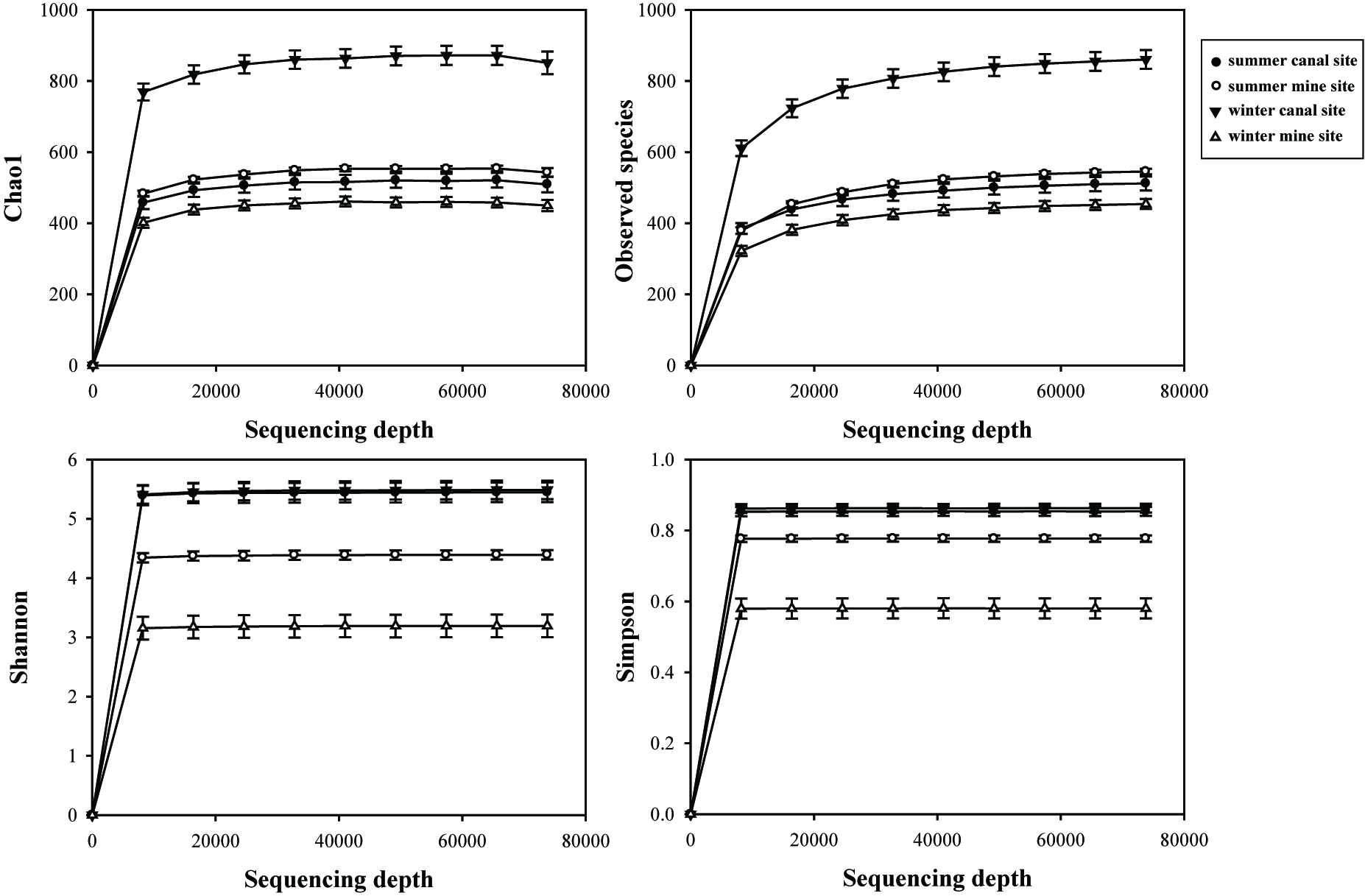


**FIG S1** Rarefaction curves for indexes of α-diversity

Supplement: Supplemental file 5 — Fig. S1. Download spectrum.00433-23-s0005.docx, DOCX file, 9.3 MB [file spectrum.00433-23-s0005.docx]
